# Identification and Quantification of Novel Major Metabolites of the Steroidal Aromatase Inhibitor, Exemestane

**DOI:** 10.1124/dmd.118.081166

**Published:** 2018-12

**Authors:** Shaman Luo, Gang Chen, Cristina I. Truica, Cynthia C. Baird, Zuping Xia, Philip Lazarus

**Affiliations:** Department of Pharmaceutical Sciences, College of Pharmacy and Pharmaceutical Sciences, Washington State University, Spokane, Washington (S.L., G.C., Z.X., P.L.); Department of Medicine, Penn State University College of Medicine, Hershey, Pennsylvania (C.I.T., C.C.B.); and Alkali Soil Natural Environmental Science Center, Northeast Forestry University, Harbin, Heilongjiang, China (S.L.)

## Abstract

Exemestane (EXE) is an aromatase inhibitor used for the prevention and treatment of estrogen receptor–positive breast cancer. Although the known major metabolic pathway for EXE is reduction to form the active 17*β*-dihydro-EXE (17*β*-DHE) and subsequent glucuronidation to 17*β*-hydroxy-EXE-17-O-*β*-D-glucuronide (17*β*-DHE-Gluc), previous studies have suggested that other major metabolites exist for exemestane. In the present study, a liquid chromatography–mass spectrometry (LC-MS) approach was used to acquire accurate mass data in MS^E^ mode, in which precursor ion and fragment ion data were obtained simultaneously to screen novel phase II EXE metabolites in urine specimens from women taking EXE. Two major metabolites predicted to be cysteine conjugates of EXE and 17*β*-DHE by elemental composition were identified. The structures of the two metabolites were confirmed to be 6-methylcysteinylandrosta-1,4-diene-3,17-dione (6-EXE-cys) and 6-methylcysteinylandrosta-1,4-diene-17*β*-hydroxy-3-one (6-17*β*-DHE-cys) after comparison with their chemically synthesized counterparts. Both underwent biosynthesis in vitro in three stepwise enzymatic reactions, with the first involving glutathione conjugation. The cysteine conjugates of EXE and 17*β*-DHE were subsequently quantified by liquid chromatography–mass spectrometry in the urine and matched plasma samples of 132 subjects taking EXE. The combined 6-EXE-cys plus 6-17*β*-DHE-cys made up 77% of total EXE metabolites in urine (vs. 1.7%, 0.14%, and 21% for EXE, 17*β*-DHE, and 17*β*-DHE-Gluc, respectively) and 35% in plasma (vs. 17%, 12%, and 36% for EXE, 17*β*-DHE, and 17*β*-DHE-Gluc, respectively). Therefore, cysteine conjugates of EXE and 17*β*-DHE appear to be major metabolites of EXE in both urine and plasma.

## Introduction

Breast cancer is the most frequently diagnosed cancer in the United States (Howlader et al., 2016). An estimated 252,710 new cases of female breast cancer were diagnosed in the United States in 2017 with approximately 75% of these being estrogen receptor–positive (ER+) (Osborne and Schiff, 2011; Siegel et al., 2017). In postmenopausal women, treatment of early-stage ER+ breast cancer has focused primarily on the elimination of estrogen-induced tumor cell growth. Aromatase inhibitors (AIs), similar to the steroidal substrate analog exemestane (EXE), act to inhibit aromatase activity by blocking the biosynthesis of estrone and estradiol, thereby preventing estrogen-induced tumor cell growth (Miller, 1999; Campos, 2004; Santen et al., 2009). Clinical trials have shown that the use of AIs increases disease-free survival and decreases the occurrence of contralateral breast cancer as compared with the use of selective estrogen receptor modulators, such as tamoxifen (Howell et al., 2005; Ferretti et al., 2006; Eisen et al., 2008; Forbes et al., 2008). EXE is widely used for the adjuvant treatment and prevention of breast cancer in postmenopausal women (Wang and Chen, 2006; Hong et al., 2007; Deeks and Scott, 2009; Petkov et al., 2009). Although AIs such as EXE represent an improvement in treatment and prevention of breast cancer, considerable interindividual variation exists in patients’ responses to these drugs (Paridaens et al., 2003; Chia et al., 2008; Campos et al., 2009; Glück, 2010). The causes for this interindividual variability have not been clearly elucidated.

Phase I metabolites of EXE have been identified in vivo. In addition to the major active phase I metabolite 17*β*-dihydro-EXE (17*β*-DHE), several minor metabolites with much lower activities are formed, including 6*ξ*-hydroxy-6*ξ*-hydroxymethylandrosta-1,4-diene-3,17-dione; 6*ξ*-hydroxyandrosta-1,4-diene-3,17-dione; 3*ξ*-hydroxy-5*ξ*-androst-1-ene-6-methylene-17-one; 6*ξ*-17*β*-dihydroxy-6*ξ*-hydroxymethylandrosta-1,4-diene-3-one; and 6*ξ*-17*β*-dihydroxyandrosta-1,4-diene-3-one (Evans et al., 1992; Cavalcanti Gde et al., 2011; de Albuquerque Cavalcanti et al., 2011). Cytosolic aldo-keto reductase 1Cs and carbonyl reductase 1 are highly active in EXE reduction to 17*β*-DHE in vitro, and several common variants in the cytosolic keto steroid reductases have been associated with altered enzymatic activity in vitro (Platt et al., 2016; Peterson et al., 2017). Multiple hepatic monooxygenases from cytochrome P450 families 1, 2, and 3 were confirmed to catalyze the production of 6-hydroxymethylandrosta-1,4,6-triene-3,17-dione, 17α-DHE as well as the active metabolite 17*β*-DHE (Peterson et al., 2017). CYP4A11 was also found to be responsible for the formation of 17*β*-DHE, whereas CYP3A was active in EXE oxidation to form 6-hydroxymethylexemestane (Kamdem et al., 2011).

UGT2B17 is the major enzyme responsible for the glucuronidation of 17*β*-DHE, and the *UGT2B17* deletion polymorphism has been linked to increased levels of 17*β*-DHE formation in plasma of women taking EXE (Sun et al., 2010; Luo et al., 2018). Interestingly, whereas drastic decreases in 17*β*-hydroxy-EXE-17-*O*-*β*-d-glucuronide (17*β*-DHE-Gluc) levels (e.g., up to 29-fold in plasma) were associated with increasing numbers of the *UGT2B17* deletion allele in women taking EXE, only a small (1.3-fold) increase in plasma 17*β*-DHE was observed in the same women (Luo et al., 2018). This suggests that other metabolic or excretion pathways may also play a role in EXE metabolism.

The goal of the present study was to characterize other major phase II metabolites of EXE. Results are presented demonstrating the existence of novel phase II cysteine conjugate EXE metabolites in both the urine and plasma of women taking EXE.

## Materials and Methods

### 

#### Chemicals and Materials.

EXE was purchased from Sigma-Aldrich (St. Louis, MO). 17*β*-DHE, 17*β*-DHE-Gluc, 17*β*-OH-EXE-d3 (D_3_-17*β*-DHE), 17*β*-OH-EXE-d3-17-*O*-*β*-d-glucuronide, and EXE-19-d3 (D_3_-EXE) were purchased from Toronto Research Chemicals (North York, ON, Canada). Ammonium formate was obtained from Sigma-Aldrich, whereas ammonium acetate and formic acid were purchased from Thermo Fisher Scientific (Waltham, MA). Acetonitrile was purchased from Merck (Kenilworth, NJ). Solvent and buffer modifiers for ultra-pressure liquid chromatography–mass spectrometry (UPLC-MS) analysis including acetonitrile, ammonium formate, ammonium acetate, and formic acid were all liquid chromatography–mass spectrometry (LC-MS) grade. Milli-Q water (Sigma, St. Louis, MO) was used for the preparation of all solutions. Pooled human liver cytosol (HLC; mixed gender, pool of 50 subjects) was purchased from XENOTECH (Kansas City, KS). Reduced l-glutathione (GSH) and glutamyltranspeptidase (*γ*-GT) from equine kidney were purchased from Sigma-Aldrich. l-Cysteine was purchased from Alfa Aesar (Heysham, Lancashire, UK). Preparative C-18 thin-layer chromatography (TLC) plates (20 cm × 20 cm × 1 mm) were obtained from Sigma-Aldrich. Hexane, diethyl ether, acetonitrile, potassium hydroxide, and methanol used for chemical synthesis were of American Chemical Society reagent grade or higher quality and were purchased from VWR (Radnor, PA).

#### Subjects and Samples.

One hundred and thirty-two postmenopausal breast cancer patients (one Hispanic, two African Americans, two Asians, and 127 Caucasians; age range: 35–89 years) with ER+ breast tumors were recruited into this study from the Breast Oncology Clinic at the Penn State Hershey Cancer Institute. Approval was obtained from the Institutional Review Board at Penn State University with informed consent obtained from all subjects. Subjects took a single pill (25 mg) of EXE daily (orally) for at least 28 consecutive days and provided blood (10 ml) and urine (up to 50 ml) specimens between 4 and 6 hours after pill ingestion as described previously (Luo et al., 2018). Blood was separated by centrifugation at 1300*g* for 15 minutes at room temperature. Aliquoted plasma and buffy coat fractions of blood samples and aliquoted urine samples were stored at −80°C until analysis. As a control for EXE metabolism, specimens obtained from women not taking EXE who were recruited into other studies at Penn State University College of Medicine (Hershey, PA; *n* = 10) were also examined to exclude false positives (Ashmore et al., 2013).

#### Sample Preparation for Identification of EXE Metabolites.

Ten urine specimens from subjects taking EXE and 10 urine specimens from control subjects who did not take EXE were selected for analysis. One hundred microliters of 100% methanol was added to a 50-*µ*l aliquot of each urine sample to extract EXE and its metabolites. After vortexing and subsequent centrifugation at 16,100*g* for 10 minutes at 4°C, the supernatant was transferred to a fresh sample vial for analysis by UPLC-MS.

#### UPLC-MS Conditions for Screening of EXE Metabolites in Urine.

For the simultaneous identification of potential novel phase II metabolites and analysis of known metabolites (including EXE, 17*β*-DHE, and 17*β*-DHE-Gluc), urine samples were prepared as described earlier and analyzed using a UPLC-MS system (Waters, Milford, MA) consisting of an ACQUITY UPLC pump, an ACQUITY FTN (flow through needle) sample manager, an ACQUITY UPLC BEH column C18 (2.1 × 100 mm, 1.7-*µ*m particle size), and a XEVO G2-S Quadrupole Time of Flight (QTOF) mass spectrometer. UPLC was performed at a flow rate of 0.4 ml/min with solvent A (5 mM ammonium formate and 0.01% formic acid in water) and solvent B (100% acetonitrile) using the following conditions for urine specimens: 0.5 minutes at 25% solvent B, a linear gradient to 100% solvent B in 4 minutes, 1.5 minutes at 100% solvent B followed by re-equilibrium with 25% solvent B for 2 minutes. The injection volume of each prepared urine sample was 2 *µ*l. The column temperature was 35°C. The Waters XEVO G2-S QTOF MS was equipped with an electrospray ionization probe operated in the positive-ion mode, with a capillary voltage at 0.6 kV. Nitrogen was used as both the cone and desolvation gases with flow rates maintained at 50 and 800 l/h, respectively. Ultra-pure argon was used as the collision gas with a flow rate of 0.1 l/h. The source and desolvation gas temperatures were 120°C and 500°C, respectively, and the dwell time for each ion was 300 ms. The mass spectrometer was operated in MS^E^ mode (MassLynx; Waters) as a nontargeted method for metabolite identification (Plumb et al., 2006). In this method, two interleaved scan functions were used for data acquisition, with both functions collecting data over the same mass range [(m/z)^+^ = 50–1250]. The first scan acquired data using a low collision energy and collected information on the intact (parent) ions in each sample. The second scan acquired data with ramped collision energy from low to high and collected the fragment (daughter) ion data of the ions in the preceding scan (Bateman et al., 2007).

#### Chemical Synthesis of 6-Methylcysteinylandrosta-1,4-diene-3,17-dione and 6-Methylcysteinylandrosta-1,4-diene-17*β*-hydroxy-3-one.

The EXE and 17*β*-DHE used for the chemical synthesis of 6-methylcysteinylandrosta-1,4-diene-3,17-dione (6-EXE-cys) and 6-methylcysteinylandrosta-1,4-diene-17*β*-hydroxy-3-one (6-17*β*-DHE-cys) were synthesized as previously described (Platt et al., 2016). Nuclear magnetic resonance (NMR) spectra were recorded with a Bruker (Billerica, MA) Avance I instrument with 500 MHz for hydrogen and 125 MHz for carbon. The abbreviations for NMR data are the following: s, singlet; d, doublet; dd, doublet of doublets; m, multiplet; J, coupling constant; δ, chemical shift.Chemical shifts were measured based on the residual protium in NMR solvent, and product purity was determined by UPLC spectrum monitored at 254 nm.

1. *6-EXE-cys*. EXE (8.0 mg, 0.027 mmol) and l-cysteine (9.7 mg, 0.080 mmol) were added to a 10-ml round-bottom flask under the protection of argon, followed by the addition of degassed 1.25 N potassium hydroxide in 25% methanol (1 ml). The mixture was stirred at ambient temperature for 24 hours, and the pH was then adjusted to 5.0 with 2 N cold HCl. The reaction mixture was subsequently applied on a preparative C-18 reverse-phase TLC plate developing in a chamber containing 20% acetonitrile in water. The product band determined by 254-nm UV visualization at R_f_ = 0.3 was scratched off from the TLC plate with a spatula into a clean 100-ml round-bottom flask. Pure methanol (40 ml) was added to extract the product. After filtration of the methanol-extracted mixture through a fritted funnel and flushing with methanol, the solvent was removed by a rotary evaporator. The residue was washed with 100% hexane (2 × 1 ml) and 100% diethyl ether (2 × 1 ml) and dried to afford the product (1.5 mg, Y = 13%) as an off-white semisolid [^1^H NMR (CD_3_OD): *δ* 7.31 (d, J = 10.2 Hz, 1 H), 6.23 (dd, J_1_ = 10.2 Hz, J_2_ = 1.8 Hz, 1 H), 6.12 (m, 1 H), 3.71 (m, 1 H), 3.16–3.19 (m, 1 H), 2.93–3.03 (m, 2 H), 2.83 (m, 1 H), 2.73 (m, 1 H), 2.46 (m, 1 H), 2.35 (m, 1 H), 1.95–2.12 (m, 3 H), 1.88 (m, 1 H), 1.64–1.84 (m, 3 H), 1.25–1.39 (m, 3 H), 1.33 (s, 3 H), 1.06–1.12 (m, 1 H), 0.98 (s, 3 H); ^13^C DEPT135 (CD_3_OD): *δ* 159.6, 127.0, 122.0, 55.0, 51.2, 40.7, 39.9 (CH_2_), 36.2 (CH_2_), 35.9, 34.6 (CH_2_), 32.1 (CH_2_), 23.2 (CH_2_), 22.6 (CH_2_), 18.9, 14.0]. The purity was >95%.
2. *6-17β-DHE-cys*. 17*β*-DHE (8.4 mg, 0.028 mmol) and l-cysteine (9.9 mg, 0.082 mmol) were added to a 10-ml round-bottom flask in a reaction procedure identical to that described earlier for 6-methylcysteinylandrosta-1,4-diene-3,17-dione. The final residue was also washed with hexane (2 × 1 ml) and diethyl ether (2 × 1 ml) and dried to afford the product (1.5 mg, Y = 13%) as a white semisolid [^1^H NMR (CD_3_OD): *δ* 7.31 (d, J = 10.2 Hz, 1 H), 6.23 (dd, J_1_ = 10.2 Hz, J_2_ = 1.8 Hz, 1 H), 6.10 (m, 1 H), 3.70 (m, 1 H), 3.57 (dd, J_1_ = J_2_ = 8.5 Hz, 1 H), 3.17 (m, 1 H), 2.91–2.99 (m, 2 H), 2.80 (m, 1 H), 2.70 (dd, J_1_ = 12.7 Hz, d_2_ = 6.5 Hz, 1 H), 2.22 (ddd, J_1_ = 12.4 Hz, J_2_ = J_3_ = 3.9 Hz, 1 H), 1.96–1.99 (m, 1 H), 1.71–1.90 (m, 4 H), 1.68 (m, 1 H), 1.63 (m, 2 H), 1.42–1.53 (m, 2 H), 1.31 (s, 3 H), 0.9–1.2 (m, 5 H), 0.83 (s, 3 H); ^13^C DEPT135 (CD_3_OD): *δ* 160.1, 126.8, 121.8, 81.8, 55.4 (2 C), 55.3, 51.0, 40.9 (CH_2_), 40.8, 37.4 (CH_2_), 36.5, 34.7 (CH_2_), 30.3 (CH_2_), 24.2 (CH_2_), 23.6 (CH_2_), 18.9, 11.4]. The purity was >95%.

#### Biosynthesis of 6-EXE-cys, 6-17*β*-DHE-cys, and Deuterium-Labeled Internal Standards.

6-EXE-cys, 6-17*β*-DHE-cys, and their corresponding deuterium-labeled internal standards were biosynthesized in a three-step process involving an initial GSH conjugation reaction of EXE or 17*β*-DHE, removal of glutamic acid from the GSH conjugates using purified active *γ*-GT, and subsequent removal of glycine from the cysteinylglycine conjugate using HLC as a source of dipeptidase enzyme.

1. *GSH conjugation of EXE and 17β-DHE*. Pooled HLC (1 mg) was added to a reaction containing 100 mM potassium phosphate (pH = 7.4) and 250 *μ*M EXE or 335 *μ*M 17*β*-DHE in a total volume of 1 ml. The reaction mixture was preincubated at 37°C for 3 minutes prior to the addition of 50 *µ*l of 100 mM GSH (final GSH concentration = 5 mM) (Lash et al., 1999; Zarth et al., 2015; Shi et al., 2016). After 2 hours at 37°C, the reaction was stopped with an equal reaction volume of ice-cold acetonitrile and centrifuged at 16,100*g* for 10 minutes at 4°C. Aliquots (50 *µ*l) of the supernatant were injected onto the ACQUITY UPLC BEH C18 column (2.1 × 100 mm, 1.7-*µ*m particle size) for separation by UPLC. The UPLC conditions were the same as those described earlier for screening of EXE metabolites. Conjugate-containing fractions were collected at UPLC retention times of 0.8–2.0 minutes for the EXE-GSH conjugate or 0.5–2.0 minutes for the 17*β*-DHE-GSH conjugate prior to their concentration and removal of organic solvent by speedvac.
2. *γ-GT–mediated removal of γ-glutamyl from the GSH conjugates of EXE and 17β-DHE*. The EXE-GSH and 17*β*-DHE–GSH conjugates collected earlier were digested at 37°C for 15 minutes with 1.0 U/ml *γ*-GT, 100 mM potassium phosphate (pH 7.4), 20 mM glycylglycine, and 5 mM dithiothreitol in a total volume of 600 *µ*l as previously described (Lash et al., 1999; Del Corso et al., 2006; Grillo et al., 2008). The reaction was stopped with an equal reaction volume of ice-cold acetonitrile and centrifuged at 16,100*g* for 10 minutes at 4°C, and aliquots (50 *µ*l) of the supernatant were injected onto the same UPLC system described earlier. Conjugate-containing fractions were collected at UPLC retention times of 1.0–1.5 minutes for the EXE-cysteinylglycine conjugate and 0.5–1.0 minute for the 17*β*-DHE–cysteinylglycine conjugate prior to their concentration to half their original volume by speedvac.
3. *Dipeptidase-mediated removal of glycine from cysteinylglycine conjugates*. Collected EXE-cysteinylglycine or 17*β*-DHE–cysteinylglycine conjugates were digested with 1.0 mg/ml pooled HLC in 100 mM potassium phosphate buffer (pH 7.4), 0.2 mM MnCl_2_, and 5 mM dithiothreitol at 37°C in a final reaction volume of 200 *µ*l (Hirota et al., 1986; Josch et al., 1998; Cappiello et al., 2004; Del Corso et al., 2006). The reaction was stopped with an equal reaction volume of ice-cold acetonitrile and centrifuged at 16,100*g* for 10 minutes at 4°C, and aliquots (50 *µ*l) of the supernatant were injected onto the same UPLC system described earlier.
4. *Biosynthesis of D_3_-6-EXE-cys and D_3_-6-17β-DHE-cys*. In enzymatic reactions identical to those described in steps 1–3, D_3_-EXE or D_3_-17*β*-DHE was used as the starting material at 330 *μ*M in 1-ml reactions. Collected D_3_-6-EXE-cys and D_3_-6-17*β*-DHE-cys conjugates were dried by speedvac and dissolved in pure methanol. The concentrations for the stock solutions of D_3_-6-EXE-cys and D_3_-6-17*β*-DHE-cys, estimated by comparing MS peak area with the chemically synthesized standard, were 2.5 and 10 *μ*g/ml, respectively.

#### Sample Preparation for Quantification of EXE and Its Metabolites in Urine and Plasma.

To quantify in vivo levels of EXE and its metabolites, the sample preparation method was modified from that described earlier for metabolite identification. For EXE metabolite analysis in plasma, a 2.5-*µ*l aliquot of each plasma sample was first spiked with 2.5 *µ*l of a mixture of deuterium-labeled internal standards in methanol [D_3_-EXE (0.17 *μ*M), D_3_-17*β*-DHE (1.7 *μ*M), 17*β*-OH-EXE-d3-17-*O*-*β*-d-glucuronide (0.52 *μ*M), D_3_-6-EXE-cys (0.06 *μ*M), and D_3_-6-17*β*-DHE-cys (0.25 *μ*M)]. Twenty microliters of pure methanol was then added to extract EXE and its metabolites and to precipitate proteins. After vortexing and subsequent centrifugation at 16,100*g* for 10 minutes at 4°C, 15 *µ*l of the supernatant was transferred to a sample vial and mixed with 15 *µ*l of water prior to analysis by UPLC-MS.

For EXE metabolite analysis in urine, a 2.5-*µ*l aliquot of each urine sample was first spiked with 2.5 *µ*l of the same deuterium-labeled internal standard mixture described earlier. Ten microliters of 75% methanol was then added to extract EXE and its metabolites. After vortexing and subsequent centrifugation at 16,100*g* for 10 minutes at 4°C, 10 *µ*l of the supernatant was transferred to a sample vial and mixed with 5 *µ*l of water prior to analysis by UPLC-MS.

#### UPLC-MS Conditions for Quantification of EXE and Its Metabolites in Urine and Plasma.

For the simultaneous quantification of EXE, 17*β*-DHE, 17*β*-DHE-Gluc, 6-EXE-cys, and 6-17*β*-DHE-cys, urine and plasma samples were prepared as described earlier. Using the same UPLC-MS system described earlier, UPLC was performed with solvent A (5 mM ammonium formate and 0.01% formic acid in water) and solvent B (100% acetonitrile) using the following conditions for both urine and plasma specimens: 0–1.5 minutes at 25% solvent B, a linear gradient to 52% solvent B for 1.5–2.5 minutes, 2.5–4.5 minutes at 52% solvent B, a linear gradient to 95% solvent B from 4.5 to 5 minutes, 5–9.5 minutes at 95% solvent B, followed by a linear gradient to initial conditions of 25% solvent B from 9.5 to 10 minutes. This was followed by a 1-minute run at 25% solvent B to equilibrate the column to initial conditions before the next sample injection. The flow rate was 0.4 ml/min from 0 to 5 minutes, 0.8 ml/min from 5 to 9.5 minutes, and back to 0.4 ml/min from 9.5 to 10 minutes. The injection volume of each prepared urine and plasma sample was 5 *µ*l. The column temperature was 35°C. The Waters XEVO G2-S QTOF MS was operated in tandem mass spectrometry (MS/MS) mode, with the electrospray ionization probe operated in the positive-ion mode and a capillary voltage at 0.6 kV. The cone and desolvation gas flow rates were maintained at 50 and 800 l/h, respectively, and the collision gas flow rate was 0.1 l/h. The source and desolvation gas temperatures were 120°C and 500°C, respectively, and the dwell time for each ion was 300 ms. The ion-related parameters for the 10 transitions monitored are listed in Table 1.

**TABLE 1:** MS/MS transitions and ion optic parameters for EXE and EXE metabolites

	ES+ MS/MS Transition	Cone Voltage	Collision Energy
	*(m/z)+*	*V*	*eV*
EXE	297.19 > 297.19	25	10
D_3_-EXE	300.20 > 300.20	25	10
17*β*-DHE	299.20 > 299.20	25	10
D_3_-17*β*-DHE	302.22 > 302.22	25	10
17*β*-DHE-Gluc	475.23 > 281.19	20	15
D_3_-17*β*-DHE-Gluc	478.25 > 284.21	20	15
6-EXE-cys	418.21 > 297.19	15	15
D_3_-6-EXE-cys	421.22 > 300.20	15	15
6-17*β*-DHE-cys	420.22 > 299.20	15	15
D_3_-6-17*β*-DHE-cys	423.24 > 302.22	15	15

D_3_-17*β*-DHE-Gluc, 17*β*-OH-EXE-d3-17-*O*-*β*-d-glucuronide.

The limits of quantification for EXE, 17*β*-DHE, 17*β*-DHE-Gluc, 6-EXE-cys, and 6-17*β*-DHE-cys were 2.1, 1.6, 1.2, 0.7, and 7.2 nM in plasma, respectively, and 2.1, 1.6, 6.3, 1.5, and 7.2 nM in urine, respectively. Standard curves were constructed by plotting the ratio of analyte peak area to peak area of the corresponding internal standard (described earlier) versus concentration of analyte standard. The concentrations of stock standards were 1000 ppm. A serial dilution of standards at concentrations ranging from 1.6 nM to 1.7 *μ*M, 1.6 nM to 1.7 *μ*M, 1.0 nM to 2.1 *μ*M, 0.7 nM to 12 *μ*M, and 4.6 nM to 2.4 *μ*M was used to establish standard curves for EXE, 17*β*-DHE, 17*β*-DHE-Gluc, 6-EXE-cys, and 6-17*β*-DHE-cys, respectively. Analyte concentrations were determined by measuring the peak area ratios of analyte to internal standard and then calculating analyte concentration from the appropriate standard curve using the Waters TargetLynx software. Urinary creatinine was measured as previously described (Luo et al., 2018).

#### Statistical Analysis.

Calculations of mean and S.E. were performed using Prism (version 7; GraphPad Software, San Diego, CA).

## Results

### 

#### Identification of EXE and 17*β*-DHE Conjugates.

Urine specimens from subjects taking EXE were extracted and analyzed by UPLC-MS using MS^E^, a nontargeted method for metabolite identification that allows for the scanning of both intact ions (channel 1) and fragment ions (channel 2) simultaneously over the same mass range.

**Fig. 1.**
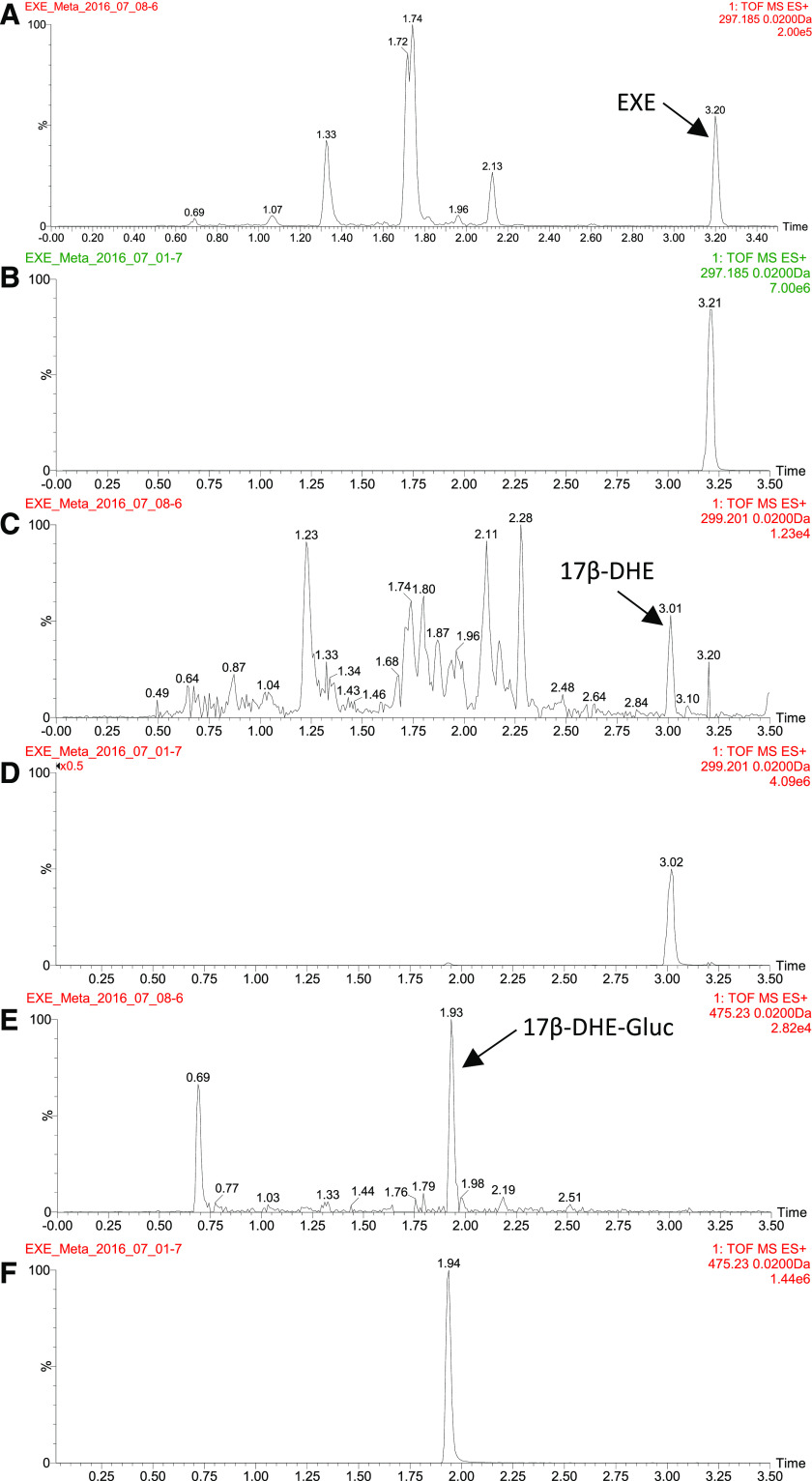
UPLC/MS^E^ analysis of EXE metabolites in urine from a subject taking EXE (A, C, and E) versus chemical standards (B, D, and F). (A and B) Extracted ion chromatogram of 297.185 (EXE) from channel 1 (intact ion). (C and D) Extracted ion chromatogram of 299.201 (17*β*-DHE) from channel 1. (E and F) Extracted ion chromatogram of 475.23 (17*β*-DHE-Gluc) from channel 1. TOF, Time of Flight.

As shown by representative chromatographs in Fig. 1, the MS^E^ method applied in this study was sensitive enough to detect known EXE metabolites extracted from the intact ion channel 1 including EXE (Fig. 1A; extracted mass = 297.19), 17*β*-DHE (Fig. 1C; extracted mass = 299.20), and 17*β*-DHE-Gluc (Fig. 1E; extracted mass = 475.23) in the urine from subjects taking EXE. All peaks corresponded to those observed for EXE, 17*β*-DHE, and 17*β*-DHE-Gluc standards (Fig. 1, B, D, and F, respectively); none were observed in the urine of subjects not taking EXE (results not shown).

**Fig. 2.**
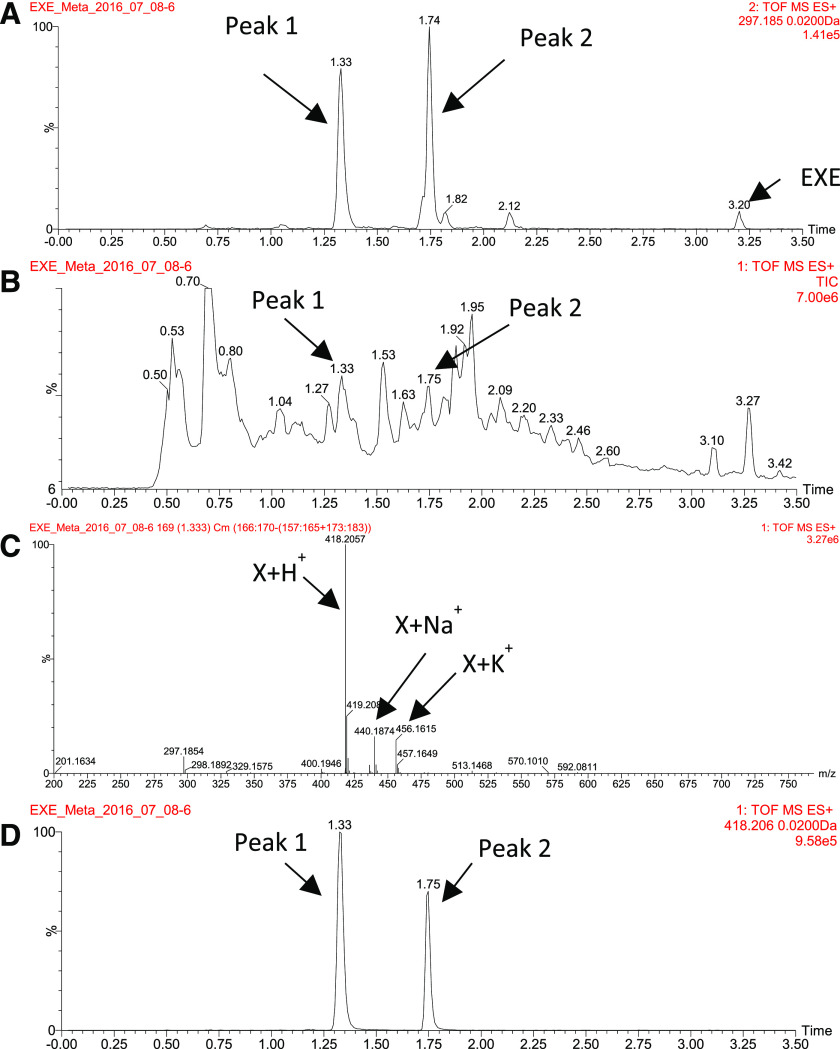
UPLC/MS^E^ analysis for identification of EXE conjugates in urine from a subject taking EXE. (A) Extracted ion chromatogram of 297.185 from channel 2 (fragment ions in screening for EXE conjugates). (B) Chromatogram of total intact ions of channel 1. (C) Mass spectrum for peak 1 in (B). (D) Extracted ion chromatogram of 418.206 from channel 1. TIC, Total Ion Current; TOF, Time of Flight.

In screening for novel EXE conjugates, the MS trace of (m/z)^+^ = 297.185 for [EXE+H]^+^, the common fragment ion for EXE conjugates, was extracted from fragment ion channel 2 (Fig. 2A). In addition to the expected peak for EXE (retention time 3.20 minutes), two major peaks (termed peaks 1 and 2, retention times = 1.33 and 1.74 minutes, respectively) were observed. Neither of these peaks were detected in urine specimens from control subjects not taking EXE (results not shown). Molecular ions for compounds that correspond to these two fragment peaks were then searched within the intact ion channel (channel 1); the retention times of peaks 1 and 2 in the intact (parent) ion channel 1 (Fig. 2B) matched that observed for peaks 1 and 2 in fragment ion channel 2 (Fig. 2A). The corresponding mass spectrum of the parent ion for peak 1 from the intact (parent) ion channel 1 showed a major peak with an (m/z)^+^ = 418.2060, presumably the [X+H]^+^ ion, where X refers to an unknown EXE conjugate (Fig. 2C). Two additional accurate mass peaks were observed (Fig. 2C), with (m/z)^+^ = 440.1874 and 456.1615, likely corresponding to the (m/z)^+^ for [X+Na]^+^ and [X+K]^+^, respectively. The accurate mass spectrum for peak 2 (data not shown) was identical to that observed for peak 1 (Fig. 2C), suggesting that the two peaks are isomers of the same EXE conjugate. The trace of 418.206 extracted from the intact (parent) ion scan from channel 1 (from Fig. 2C) exhibited two peaks (Fig. 2D) that matched the retention times of peaks 1 and 2 extracted from fragment ion channel 2 for (m/z)^+^ = 297.185 (Fig. 2A). Peak 1 was observed in all 10 urine specimens screened by MS^E^, whereas peak 2 was detected in seven of the 10 urine specimens.

The potential composition for the parent ion of (m/z)^+^ = 418.2057 was calculated using the Elemental Composition tool within MassLynx 4.1. Since the composition of EXE is C_20_H_24_O_2_, the molecular composition of possible EXE conjugates had to contain ≥20 carbons and ≥2 oxygens. The only candidate composition available that matched these criteria was C_23_H_32_NO_4_S [(m/z)^+^ = 418.2052], which exactly matched the predicted structure corresponding to the cysteine conjugate of EXE. Therefore, compounds responsible for peaks 1 and 2 in the urine of women taking EXE were predicted to be EXE-cys isomers (C_23_H_31_NO_4_S).

**Fig. 3.**
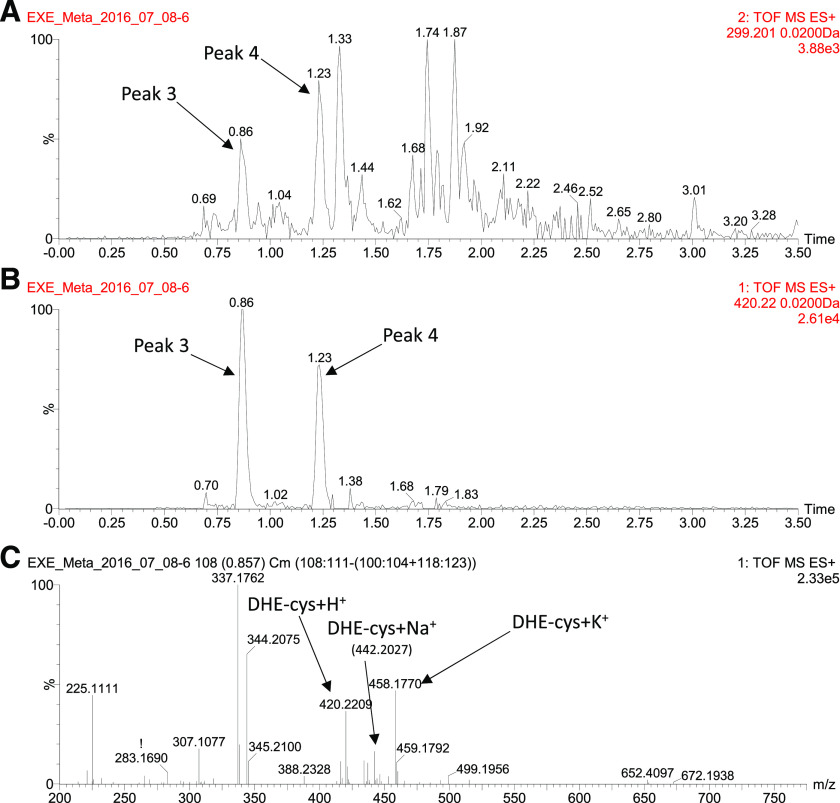
UPLC/MS^E^ analysis for identification of 17*β*-DHE conjugates from a subject taking EXE. (A) Extracted ion chromatogram of 299.201 from channel 2 (fragment ions in screening for 17*β*-DHE conjugates). (B) Extracted ion chromatogram of 420.22 from channel 1. (C) Mass spectrum for peak 3 in (B). TOF, Time Of Flight.

For identifying conjugates of 17*β*-DHE, an approach similar to that used for identifying EXE conjugates was undertaken. A trace corresponding to (m/z)^+^ = 299.201 for [17*β*-DHE +H]^+^, the common fragment ion for 17*β*-DHE conjugates, was extracted from fragment ion channel 2 (Fig. 3A), whereas a trace corresponding to the intact (parent) ion for 17*β*-DHE-cys [(m/z)^+^ = 420.221] was extracted from the intact (parent) ion channel 1 (Fig. 3B). Two fragment ion peaks were observed in the channel 2 trace (peak 3 at 0.86 minutes and peak 4 at 1.23 minutes; Fig. 3A) that aligned exactly with the retention times of peaks extracted from the intact (parent) ion [(m/z)^+^ = 420.2209] trace in channel 1 (Fig. 3B). The mass spectrum extracted for peak 3 from the intact (parent) ion channel 1 is shown in Fig. 3C. The peaks of (m/z)^+^ = 420.2209, 442.2027, and 458.1770 likely corresponded to the ions of [17*β*-DHE-cys +H]^+^, [17*β*-DHE-cys +Na]^+^, and [17*β*-DHE-cys +K]^+^, respectively. Similar to that observed for EXE-cys conjugates, peak 3 was observed in all 10 urine samples screened by the MS^E^, whereas peak 4 was observed in seven of the 10 urine samples. Again, neither of these peaks were detected in urine samples from control subjects not taking EXE (results not shown). Therefore, compounds responsible for peaks 3 and 4 were predicted to be 17*β*-DHE-cys isomers (C_23_H_33_NO_4_S).

#### Chemical Synthesis of Cysteine Conjugates of EXE and 17*β*-DHE.

To confirm the structures of the predicted cysteine conjugates of EXE and 17*β*-DHE identified earlier, 6-EXE-cys and 6-17*β*-DHE-cys were synthesized chemically. As described in the *Materials and Methods*, the structures of both chemically synthesized cysteine conjugates were confirmed by NMR, and the purity of both conjugates was confirmed by LC-MS to be >95% (results not shown).

**Fig. 4.**
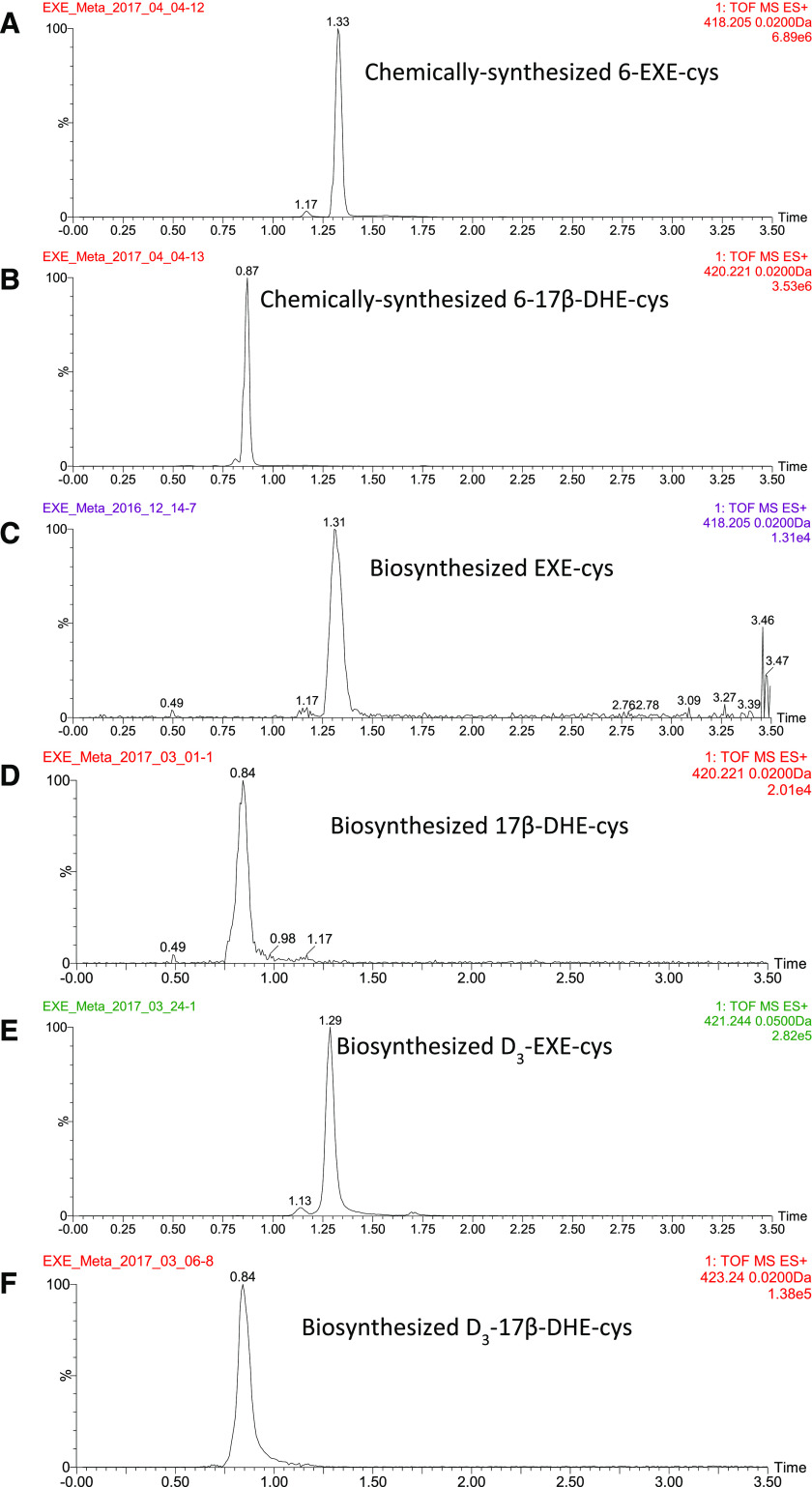
UPLC/MS^E^ analysis of chemically synthesized and biosynthesized EXE and 17*β*-DHE cysteine conjugates. (A) MS (418.205) chromatography for chemically synthesized 6-EXE-cys standard. (B) MS (420.221) chromatography for chemically synthesized 6-17*β*-DHE-cys standard. (C) MS (418.205) chromatography for biosynthesized EXE-cys. (D) MS (420.221) chromatography for biosynthesized 17*β*-DHE-cys. (E) MS (421.244) chromatography for biosynthesized D_3_-EXE-cys. (F) MS (423.240) chromatography for biosynthesized D_3_-17*β*-DHE-cys.

The retention times for peaks corresponding to chemically synthesized 6-EXE-cys and 6-17*β*-DHE-cys (Fig. 4, A and B, respectively) matched those observed for the predicted EXE-cys and 17*β*-DHE-cys peaks 1 and 3, respectively (Figs. 2D and 3B, respectively), detected in the urine of subjects taking EXE. In addition, coelution experiments were performed by adding chemically synthesized 6-EXE-cys and 6-17*β*-DHE-cys into urine specimens of EXE-treated subjects. LC-MS analysis showed increases in peak size for each compound (results not shown), further demonstrating that the urinary peaks are identical to the chemically synthesized standards. Together, these data suggest that the MS peaks 1 and 3 (Figs. 2D and 3B, respectively) from urine of women taking EXE corresponded to 6-EXE-cys and 6-17*β*-DHE-cys.

#### Biosynthesis of Cysteine Conjugates of EXE and 17*β*-DHE.

GSH is a tripeptide (*γ*-glu-cys-gly) that can conjugate to an electrophilic substrate, which can be further metabolized to form cysteine conjugates during mercapturic acid biosynthesis (Hinchman and Ballatori, 1994; van Bladeren, 2000; Hayes et al., 2005). To explore whether this mechanism of cysteine conjugate formation may occur for EXE and 17*β*-DHE in vivo, cysteine conjugates of EXE and 17*β*-DHE were enzymatically synthesized in a three-step reaction. For the synthesis of EXE-cys, the EXE-glutathione conjugate was first synthesized by incubating EXE with GSH in the presence of HLC as a source of glutathione-*S*-transferases (GSTs). Products of the reaction were analyzed by LC-MS. Three peaks (retention times = 1.15, 1.60, and 1.81 minutes) were detected as potential EXE-glutathione conjugates [(m/z)^+^ = 604.27; results not shown]. Using LC-purified peak 1 (retention time = 1.15 minutes), EXE-cys conjugates were then formed (Fig. 4C) after two additional reactions using purified *γ*-GT in reaction 1 and then HLC for reaction 2. The retention time observed for the enzymatically synthesized EXE-cys conjugate (1.31 minutes) was similar to that observed for the chemically synthesized 6-EXE-cys conjugate (1.33 minutes; Fig. 4A) and was similar to that observed for the putative 6-EXE-cys conjugate corresponding to peak 1 in the urine of subjects taking EXE (Fig. 2D).

Using 17*β*-DHE as a substrate, a similar three-step reaction approach was performed to enzymatically synthesize the 6-17*β*-DHE-cys conjugate. The retention time observed for the enzymatically synthesized 6-17*β*-DHE-cys conjugate (0.84 minutes; Fig. 4D) was similar to that observed for the chemically synthesized 6-17*β*-DHE-cys conjugate (Fig. 4B) and to that observed for the putative 17*β*-DHE-cys conjugate corresponding to peak 3 in the urine of subjects taking EXE (Fig. 3B). In addition, a similar pattern was observed for both the enzymatically synthesized D_3_-labeled 6-EXE-cys and 6-17*β*-DHE-cys conjugates (Fig. 4, E and F, respectively). All cysteine conjugates were confirmed by analysis of corresponding mass spectra (results not shown).

#### Quantification of EXE and Its Metabolites In Vivo.

**Fig. 5.**
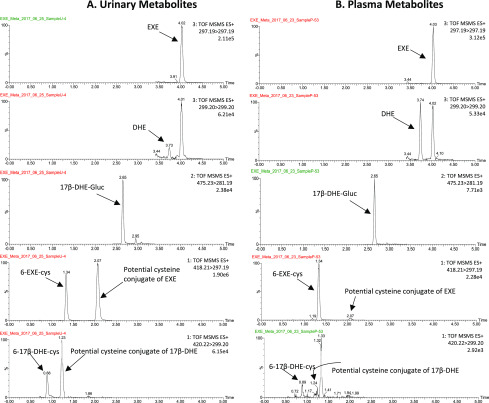
Representative chromatogram for quantification of urinary (A) and plasma (B) EXE and its metabolites in women taking EXE. MS/MS traces for quantification of EXE (297.19 > 297.19), 17*β*-DHE (299.20 > 299.20), 17*β*-DHE-Gluc (475.23 > 281.19), EXE-cys (418.21 > 297.19), and 17*β*-DHE-cys (420.22 > 299.20) in urine (A) and in plasma (B) are shown.

For quantification of EXE and its metabolites in vivo, EXE metabolites were analyzed by UPLC-MS/MS for all urine samples and matched plasma samples from 132 postmenopausal breast cancer patients who had taken 25 mg of EXE per day for at least 4 weeks. As shown for representative MS chromatograms of EXE metabolites from a subject taking EXE (Fig. 5), the retention times were 4.02 minutes for EXE, 3.73 minutes for 17*β*-DHE, 2.65 minutes for 17*β*-DHE-Gluc, 1.34 minutes for 6-EXE-cys, and 0.88 minutes for 6-17*β*-DHE-cys for both urine and plasma using this UPLC-MS/MS method. In all cases, the retention times of each peak were similar to that of their corresponding internal standard peaks (see Figs. 1 and 4). Although the predicted isomers of EXE-cys and 17*β*-DHE-cys (peaks 2 and 4 in Figs. 2D and 3B, respectively) were not quantified due to a lack of accurate standards, they may be present at similar or higher levels than that observed for 6-EXE-cys and 6-17*β*-DHE-cys if their MS response factors are similar to 6-EXE-cys and 6-17*β* -DHE-cys, respectively.

The major metabolites in the plasma of subjects treated with EXE were 17*β*-DHE-Gluc (mean = 30 nM; Table 2) and 6-EXE-cys (mean = 22 nM). The levels of 6-17*β*-DHE-cys (5.9 nM) and 17*β*-DHE (2.5 nM) were lower than that observed for the parent EXE (mean = 14 nM) in plasma, with the average percentage of 17*β*-DHE in total quantified EXE metabolites (TEM) observed at 3-fold lower levels than that observed for 17*β*-DHE-Gluc. The mean levels of the combined cysteine conjugates for plasma EXE plus 17*β*-DHE were roughly equivalent to the levels of plasma 17*β*-DHE-Gluc, with the average percentage of TEM ranging from 35% to 36% for both (6-EXE-cys + 6-17*β*-DHE-cys) and 17*β*-DHE-Gluc. The percentages of 17*β*-DHE-Gluc and 6-EXE-cys in TEM (36% and 23%, respectively) were highest when compared with other EXE metabolites in the plasma of EXE-treated subjects, ranging from 12% to 17% for 17*β*-DHE, 6-17*β*-DHE-cys, and EXE.

**TABLE 2:** Summary of EXE metabolite concentrations in plasma and urine from EXE-treated subjects (*n* = 132 subjects taking EXE)

	Plasma			Urine		
	Mean ± S.E.	Range	Percentage of TEM (Mean ± S.E.)*^a^*	Mean ± S.E.	Range	Percentage of TEM (Mean ± S.E.)*^a^*
	*nM*			*nmol/mg creatinine*		
EXE	14 ± 1.7	0–105	17 ± 0.91	0.21 ± 0.045	0–3.2	1.7 ± 0.20
17*β*-DHE	2.5 ± 0.19	0.11–14	12 ± 1.2	0.0066 ± 0.0012	0–0.099	0.14 ± 0.025
17*β*-DHE-Gluc	30 ± 4.6	0.32–358	36 ± 1.8	1.4 ± 0.37	0.0068–45	21 ± 1.6
6-EXE-cys	22 ± 2.9	0.020–226	23 ± 1.3	5.9 ± 0.69	0.0033–50	55 ± 1.6
6-17*β*-DHE-cys	5.9 ± 0.58	0–31	12 ± 0.76	1.8 ± 0.19	0.0033–10	22 ± 0.79

^a^ TEM = EXE + 17*β*-DHE + 17*β*-DHE-Gluc + 6-EXE-cys + 6-DHE-cys. The percentage of TEM was calculated for EXE or each EXE metabolite for every individual subject, with the mean then calculated for each metabolite of all 132 subjects.

Although a similar trend was observed for urinary EXE metabolites, the levels of urinary 17*β*-DHE-Gluc were 5.5-fold less than the combined cysteine conjugates for urinary EXE plus 17*β*-DHE, which were the major urinary metabolites in women taking EXE (Table 2). 17*β*-DHE-Gluc comprised, on average, 21% of total quantified urinary EXE metabolites versus the combined 6-EXE-cys plus 6-17*β*-DHE-cys conjugates, which comprised 77% of total quantified urinary EXE metabolites. The levels of urinary 17*β*-DHE-Gluc (1.4 nmol/mg creatinine) were slightly lower than those observed for 6-17*β*-DHE-cys (1.8 nmol/mg creatinine), which was 3.3-fold lower than that observed for the major urinary metabolite 6-EXE-cys. The mean levels of urinary 6-EXE-cys (mean = 5.9 nmol/mg creatinine) were almost 900-fold higher than the urinary metabolite observed at the lowest level, 17*β*-DHE (mean = 0.0066 nmol/mg creatinine).

## Discussion

**Scheme 1.**
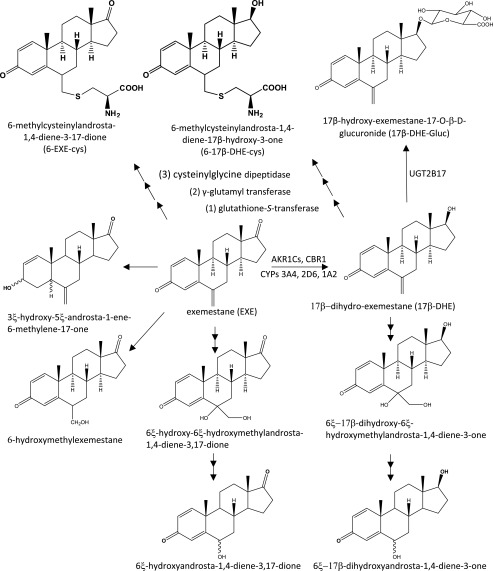
Schematic of EXE metabolism. Shown are major exemestane metabolites formed in vivo.

Previous studies indicated that the major mode of metabolism of EXE is by reduction to form 17*β*-DHE and UGT2B17-mediated glucuronidation to form 17*β*-DHE-Gluc (Sun et al., 2010; Luo et al., 2018). In the present study, two novel major EXE metabolites were identified—the cysteine conjugates of EXE (6-EXE-cys) and 17*β*-DHE (6-17*β*-DHE-cys). The mean levels of the combined 6-EXE-cys plus 6-17*β*-DHE-cys were 5.5-fold higher than those observed for 17*β*-DHE-Gluc in urine and were similar to the levels of 17*β*-DHE-Gluc observed in plasma. 6-EXE-cys formed the major cysteine conjugate of EXE, comprising 77% and 79% of the total mean cysteine conjugate levels in urine and plasma, respectively. This suggests that the formation of cysteine conjugates is the major excretion pathway for EXE in humans, with 6-EXE-cys the major urinary EXE metabolite (see Scheme 1).

Although the stereochemistry at the 6-position was not assigned in the current paper, we speculate the stereo orientation for the 6 substitute is “*α*” for both synthesized 6-EXE-cys and 6-17*β*-DHE-cys based on the proton coupling constant between the proton at the 4-position and the proton at the 6-position of both EXE-cys and 17*β*-DHE-cys conjugates. For 6-substituted steroids, long-distance coupling between H-4 and H-6 in ^1^H NMR will be observed only when the 6-substituent is in the *α* position (Chin and Warren, 1972; Schneider et al., 1973; Numazawa and Oshibe, 1994; Numazawa and Yamaguchi, 1998; Görlitzer et al., 2006). In an analysis of a series of 6-substituted phenylaliphatic steroids, Numazawa and Yamaguchi (1998) demonstrated that proton coupling between the hydrogens at the 4- and 6-positions was only observed for the 6*α*-substituted, but not the 6*β*-substituted, phenylaliphatic steroids. Similar C-4 proton signals have been reported for a series of 6-alkyl-, 6-bromo-, and 6-(bromoacetoxy)androstenediones (Numazawa and Oshibe, 1994; Numazawa and Yamaguchi, 1998). In addition, the EXE derivative 6*α*-chlormethylandrosta-1,4-dien-3,17-dion exhibits similar long-distance coupling between H-4 and H-6 (Görlitzer et al., 2006). These data are consistent with the proton coupling pattern of 6-EXE-cys and 6-17*β*-DHE-cys observed by ^1^H NMR in the present study (results not shown), suggesting that both conjugates were in the *α*-position. However, X-ray crystallography analysis of the two conjugates will provide a more definitive validation of their structure.

Previous in vitro studies in a panel of human liver microsomes demonstrated that deletion of the UGT2B17 gene resulted in significant decreases in 17*β*-DHE-Gluc formation (Sun et al., 2010). Although drastic decreases in urinary and plasma 17*β*-DHE-Gluc levels were also associated with increasing numbers of the *UGT2B17* deletion allele in women taking EXE (e.g., up to 29-fold in plasma), only a small (1.3-fold) corresponding increase in plasma 17*β*-DHE was observed in the same women (Luo et al., 2018). In the present study, 17*β*-DHE-Gluc was demonstrated to comprise 36% of the total quantified EXE metabolites in plasma, levels that were approximately equal to that observed for both of the cysteine conjugates combined (which comprised 35% of the total quantified plasma EXE metabolites) and only slightly more than that observed for 6-EXE-cys alone (which comprised 23% of total quantified plasma EXE metabolites). In addition, 17*β*-DHE-Gluc comprised only 21% of the total quantified EXE metabolites in urine. These values correspond with the fact that either no alterations or only small increases in urinary or plasma EXE and 17*β*-DHE were observed in subjects taking EXE who were homozygous for the UGT2B17 deletion polymorphism (i.e., with no active UGT2B17) in previous studies (Luo et al., 2018).

A three-step metabolism pathway similar to the first three steps of the mercapturic acid synthesis pathway was shown to be a viable in vivo mechanism of EXE and 17*β*-DHE cysteine conjugate formation in the present studies. The three steps include an initial GSH conjugation reaction catalyzed by GSTs to form EXE-GSH or 17*β*-DHE–GSH, a second reaction catalyzed by *γ*-GT to remove the glutamyl moiety from the glutathione conjugate to form EXE-cysteinylglycine or 17*β*-DHE–cysteinylglycine, and a final reaction where the glycyl moiety is removed by dipeptidase to form the EXE-cys or 17*β*-DHE-cys conjugates.

In addition to the identified 6-EXE-cys (peak 1 in Fig. 2D) and 6-17*β*-DHE-cys conjugates (peaks 3 in Fig. 3B), secondary peaks likely corresponding to EXE-cys (peak 2 in Fig. 2D) and 17*β*-DHE-cys isomers (peaks 4 in Fig. 3B) of unknown structure were observed in the urine of women taking EXE in the present study. EXE and 17*β*-DHE are *α*-*β* unsaturated ketones with several double bonds for GSH conjugation. Therefore, there are several possible positions for the initial GSH conjugation step to occur. Chemical synthesis of other potential isomers of EXE-cys and 17*β*-DHE-cys conjugates is currently underway to confirm their structures.

Similar to phase II metabolism by glucuronidation, GSH conjugation of either EXE or 17*β*-DHE likely increases their rate of excretion and eliminates their antiaromatase activities. Significant variations in levels of 6-EXE-cys and 6-17*β*-DHE-cys were observed in the urine and plasma samples of the 132 subjects. As shown in Table 2, 6-EXE-cys and 6-17*β*-DHE-cys ranged from 0.020 to 226 nM and 0 to 31 nM in plasma, respectively, and from 0.0033 to 50 nM and 0.0033 to 10 nM in urine, respectively. Interestingly, both the unidentified EXE cysteine conjugate isomer (peak 2 in Fig. 2D) and the unidentified 17*β*-DHE cysteine conjugate isomer (peak 4 in Fig. 3B) were observed in the urine of only seven of the 10 subjects examined in this study. There is significant genetic variability in the GST enzymes (Hayes et al., 2005), including common copy number variants observed for both GSTM1 and GSTT1 (Seidegard et al., 1990; Bell et al., 1993; Arruda et al., 1998; Bailey et al., 1998; Roth et al., 2000; Hayes et al., 2005). The frequencies of homozygous deletion genotypes of GSTM1 and GSTT1 are about 50% and 14% in Caucasians, and the homozygous deletion genotypes of both GSTM1 and GSTT1 were reported to be associated with risk for a variety of cancers and may be linked to alterations in drug metabolism (Hengstler et al., 1998). For example, GSTs are involved in the metabolism of azathioprine (AZA) to mercaptopurine, with 6-methylmercaptopurine riboside a major metabolite of mercaptopurine. The levels of 6-methylmercaptopurine riboside were 2-fold lower in AZA users exhibiting the GSTM1-null genotype than AZA users carrying one or two copies of GSTM1 (Broekman et al., 2018). The deletion of GSTM1 was also associated with reduced response to AZA therapy (Stocco et al., 2014). This suggests that if such GSTs are similarly involved in the metabolism of EXE, copy number variants or other functional polymorphisms could potentially play an important role in the metabolism and efficacy of EXE. Although functional polymorphisms in both the *γ*-GT and dipeptidase enzymes could similarly modify the levels of EXE-cys or 17*β*-DHE-cys observed in the plasma and/or urine of subjects taking EXE, they do not metabolize the functional parent compound, EXE, or its major active metabolite, 17*β*-DHE, and are, therefore, less likely to be important in overall patient response to EXE.

As described earlier, quantification of EXE-cys and 17*β*-DHE-cys conjugates was performed only for peaks corresponding to the known 6-cysteine conjugates (peaks 1 and 3 in Figs. 2D and 3B, respectively). Although their structure is presently unknown, other peaks corresponding to other EXE-cys and 17*β*-DHE-cys isomers were identified in the present study, potentially at levels similar to or higher than those observed for 6-EXE-cys and 6-17*β*-DHE-cys. Therefore, these conjugates likely comprise an even larger proportion of EXE metabolites in the urine and plasma of women taking EXE, further increasing the importance of cysteine conjugate formation and decreasing the overall importance of glucuronidation in the metabolism of EXE. In addition, this further supports previous results demonstrating only small changes in plasma 17*β*-DHE and no change in urinary 17*β*-DHE in subjects deficient in DHE glucuronidation capacity since the glucuronide comprises a relatively low percentage of EXE metabolites in vivo (Luo et al., 2018).

A potential limitation of the present study was that the study subjects examined were primarily Caucasians (only five non-Caucasians out of 132 female subjects). Although we did not find observable differences in the levels of EXE or its metabolites between the Caucasian subjects and the five non-Caucasian subjects examined in this study, it is possible that the patterns observed for Caucasian females may not be 100% generalizable to all populations. Additional studies of EXE metabolism in other racial groups will be necessary to better examine this. Another potential limitation of the current study was that the intermediate products of the first and second steps for the proposed three-step pathway for EXE-cys and 17*β*-DHE-cys formation were not detected in urine samples, suggesting that they are at low levels if present. This also suggests that the final two intermediate enzymatic reaction steps with *γ*-glutamyl transferase and cysteinylglycine dipeptidase to form the EXE-cys and 17*β*-DHE-cys conjugates are highly efficient.

In conclusion, two novel EXE phase II metabolites were identified in vivo in women taking EXE. The two metabolites—cysteine conjugates of EXE and 17*β*-DHE—are the major metabolites of EXE found in the urine of subjects taking EXE, comprising, on average, 77% of total quantified urinary EXE metabolites, and were at levels similar to that observed for the other major EXE metabolite, 17*β*-DHE-Gluc, in the plasma of the same subjects. Although their exact structure is presently unknown, other EXE and 17*β*-DHE cysteine conjugates were also identified, further supporting cysteine conjugate formation as the major metabolism pathway for EXE in vivo. The pathways involved in EXE-cys or 17*β*-DHE-cys formation could, therefore, potentially play an important role in the pharmacokinetics and pharmacodynamics of EXE.

## Abbreviations

AI: aromatase inhibitor
AZA: azathioprine
D_3_-17*β*-DHE: 17*β*-OH-EXE-d3
D_3_-EXE: EXE-19-d3
17*β*-DHE: 17*β*-dihydro-EXE
6-17*β*-DHE-cys: 6-methylcysteinylandrosta-1,4-diene-17*β*-hydroxy-3-one
17*β*-DHE-Gluc: 17*β*-hydroxy-EXE-17-*O*-*β*-d-glucuronide
ER+: estrogen receptor–positive
EXE: exemestane
6-EXE-cys: 6-methylcysteinylandrosta-1,4-diene-3,17-dione
GSH: l-glutathione
GST: glutathione-*S*-transferase
*γ*-GT: glutamyltranspeptidase
HLC: human liver cytosol
LC-MS: liquid chromatography–mass spectrometry
MS/MS: tandem mass spectrometry
QTOF: Quadrupole Time of Flight
TEM: total quantified EXE metabolite
TLC: thin-layer chromatography
UPLC-MS: ultra-pressure liquid chromatography–mass spectrometry
s: singlet
d: doublet
dd: doublet of doublets
m: multiplet
J: coupling constant
δ: chemical shift
